# The absence of AQP4/TRPV4 complex substantially reduces acute cytotoxic edema following ischemic injury

**DOI:** 10.3389/fncel.2022.1054919

**Published:** 2022-12-08

**Authors:** Petra Sucha, Zuzana Hermanova, Martina Chmelova, Denisa Kirdajova, Sara Camacho Garcia, Valeria Marchetti, Ivan Vorisek, Jana Tureckova, Eyar Shany, Daniel Jirak, Miroslava Anderova, Lydia Vargova

**Affiliations:** ^1^Second Faculty of Medicine, Charles University, Prague, Czechia; ^2^Department of Cellular Neurophysiology, Institute of Experimental Medicine of the CAS, Prague, Czechia; ^3^Department of Diagnostic and Interventional Radiology, Institute of Clinical and Experimental Medicine, Prague, Czechia; ^4^First Faculty of Medicine, Institute of Biophysics and Informatics, Charles University, Prague, Czechia

**Keywords:** TRPV4, AQP4, ECS diffusion, MRI, cerebral ischemia, brain edema

## Abstract

**Introduction:**

Astrocytic Aquaporin 4 (AQP4) and Transient receptor potential vanilloid 4 (TRPV4) channels form a functional complex that likely influences cell volume regulation, the development of brain edema, and the severity of the ischemic injury. However, it remains to be fully elucidated whether blocking these channels can serve as a therapeutic approach to alleviate the consequences of having a stroke.

**Methods and results:**

In this study, we used *in vivo* magnetic resonance imaging (MRI) to quantify the extent of brain lesions one day (D1) and seven days (D7) after permanent middle cerebral artery occlusion (pMCAO) in AQP4 or TRPV4 knockouts and mice with simultaneous deletion of both channels. Our results showed that deletion of AQP4 or TRPV4 channels alone leads to a significant worsening of ischemic brain injury at both time points, whereas their simultaneous deletion results in a smaller brain lesion at D1 but equal tissue damage at D7 when compared with controls. Immunohistochemical analysis 7 days after pMCAO confirmed the MRI data, as the brain lesion was significantly greater in AQP4 or TRPV4 knockouts than in controls and double knockouts. For a closer inspection of the TRPV4 and AQP4 channel complex in the development of brain edema, we applied a real-time iontophoretic method *in situ* to determine ECS diffusion parameters, namely volume fraction (α) and tortuosity (λ). Changes in these parameters reflect alterations in cell volume, and tissue structure during exposure of acute brain slices to models of ischemic conditions *in situ*, such as oxygen-glucose deprivation (OGD), hypoosmotic stress, or hyperkalemia. The decrease in α was comparable in double knockouts and controls when exposed to hypoosmotic stress or hyperkalemia. However, during OGD, there was no decrease in α in the double knockouts as observed in the controls, which suggests less swelling of the cellular components of the brain.

**Conclusion:**

Although simultaneous deletion of AQP4 and TRPV4 did not improve the overall outcome of ischemic brain injury, our data indicate that the interplay between AQP4 and TRPV4 channels plays a critical role during neuronal and non-neuronal swelling in the acute phase of ischemic injury.

## Introduction

During ischemic injury, the insufficient blood supply to the brain tissue leads to a lack of oxygen and nutrients and, subsequently, to the accumulation of harmful substances that often results in cell death and is accompanied by massive brain edema. Generally, two types of cerebral edema are described, vasogenic (extracellular) and cytotoxic (intracellular). The vasogenic edema results from a disruption of blood brain barrier (BBB) and its tight junctions and from subsequent extravasation of blood proteins and fluid into extracellular space (ECS). On the contrary, cytotoxic edema is caused by the disruption of ion homeostasis and its main characteristic is excessive cellular swelling (Ho et al., 2012). Although it is understood that astrocytes play a major role in cytotoxic brain edema formation (Liang et al., 2007), it was demonstrated that neurons also markedly and rapidly change their volume within seconds in response to brain ischemia. This is manifested as swelling of the neuronal somas as well as dendritic “beading” (Steffensen et al., 2015; Hellas and Andrew, 2021). However, neuronal swelling does not appear simply as an osmotic event but is elicited by spreading depolarization, which occurs during ischemic brain injury. It can also be caused by neurotoxins, which artificially increase the intracellular concentration of Na^+^ (Hellas and Andrew, 2021). On the contrary, glial swelling during ischemia results directly from the osmotic imbalance caused by spreading membrane depolarization and the subsequent accumulation of ions, glutamate, reactive oxygen species, and other harmful substances (Risher et al., 2009; Song and Yu, 2014). Osmotic cellular swelling is then counteracted by the process of a regulatory volume decrease (RVD). Volume recovery of the cells during RVD is achieved by the efflux of cytosolic K^+^, Cl^–^ and other intracellular osmolytes, which is accompanied by water movement from the cells into the ECS (Pasantes-Morales and Morales Mulia, 2000; Pasantes-Morales et al., 2000; Ordaz et al., 2004; Wilson and Mongin, 2018). In the literature, studies performed on mature neurons do not support the idea of RVD, and this phenomenon is thus mostly attributed to glia, especially to astrocytes (Mola et al., 2016; Wilson and Mongin, 2018; Hellas and Andrew, 2021; Reed and Blazer-Yost, 2022).

The process of volume regulation is associated with numerous membrane channels and transporters, including volume-regulated anion channels (VRACs), Aquaporin 4 (AQP4) channels, Transient receptor potential 4 (TRPV4) channels, Inwardly rectifying K^+^4.1 channels (Kir4.1) (Mola et al., 2021; Reed and Blazer-Yost, 2022) or Swelling-activated chloride channels (LRRC8A) (Formaggio et al., 2019). AQP4 channels represent the main water pathway and they cluster on the perivascular membranes of astrocytes and retinal macroglia (Wolburg et al., 2011; Nagelhus and Ottersen, 2013).

Water transport through AQP4 is bi-directional and depends on the osmotic gradients between ECS and astrocytic cytosol. It was previously demonstrated that the role of AQP4 channels in brain edema development differs based on the type of edema involved. The AQP4-deficient mice are relatively safe from water influx during cytotoxic edema development, however they show worsened outcome of vasogenic cerebral edema, because the water drainage from brain parenchyma is compromised (Papadopoulos and Verkman, 2007; Clement et al., 2020). It has been stated that AQP4 channels form a functional complex with TRPV4 channels, which play a crucial role in RVD and glial volume regulation in general. In this complex, TRPV4 channels mediate cell response to the membrane stretch caused by the excessive water influx during cytotoxic edema development and to mechanical stimuli in general (Liedtke, 2007; Redmon et al., 2021) (Liedtke, 2007; Redmon et al., 2021). The activation of TRPV4 channels increases intracellular Ca^2+^ concentration, which triggers RVD and leads to water efflux *via* the AQP4 channels and, therefore, strongly affects the cellular response to the development of cytotoxic brain edema (Benfenati et al., 2011; Jo et al., 2015). In addition, recent study showed correlation between the increased expression of AQP4/TRPV4 complex and larger peri-tumor edema in patients (Faropoulos et al., 2021).

Changes in cell volume during their swelling or RVD are compensated for by a reciprocal change of the ECS volume, followed by changes in the ECS diffusion properties, leading to a higher concentration of potentially neurotoxic substances; this may be an additional factor contributing to the tissue damage (Sykova and Nicholson, 2008). Brain ECS not only represents an essential microenvironment surrounding brain cells, but also an important route for inter-cellular communication and the transport of metabolites and chemicals (Vizi et al., 2004; Sykova and Vargova, 2008). The diffusion parameters of the ECS, extracellular volume fraction α and tortuosity λ, govern the extracellular diffusion of neuroactive substances and also reflect fairly accurately the current structure of the brain (Thorne and Nicholson, 2006; Anderova et al., 2011; Nicholson and Hrabetova, 2017). Our previous findings revealed that changes in the ECS volume fraction α do not always correlate with astrocyte volume changes (Anderova et al., 2014) since the ECS volume alterations measured by the real-time iontophoretic (RTI) method reciprocally reflect the volume changes in all cellular elements of the tissue, i.e., in both neurons and glial cells.

Both AQP4 and TRPV4 channels are involved in brain water and ion homeostasis, edema development, and cell volume regulation, and thus manipulation of their expression affects the impact of experimental cerebral ischemia. However, their deletion or block does not appear to have a clear protective or deleterious effect, and the available data are quite controversial depending on the ischemic model used and the method used to quantify the extent of the damage. In this study we decided to examine not only the effect of the deletion of individual channels (AQP4^–/–^ and TRPV4^–/–^) but, more importantly, of both channels together (AQP4^–/–^/TRPV4^–/–^). We performed magnetic resonance imaging (MRI) *in vivo* to assess the impact of permanent middle cerebral artery occlusion (pMCAO) on the size of ischemic brain damage in all experimental groups, and the MRI results were verified by immunohistochemistry. For a closer inspection of the function of AQP4 and TRPV4 in edema development, we implemented the RTI method *in situ* to clarify to what extent the deletion of both AQP4 and TRPV4 channels can induce changes in the ECS volume fraction and tortuosity when cell swelling is evoked by hypoosmotic stress, hyperkalemia or oxygen-glucose deprivation (OGD). Since RTI and MRI methods both reflect alterations in all cellular elements, we used in the general statement term non-neuronal cells to all cell types except from neurons, while terms neurons, astrocytes, microglia, or oligodendrocyte precursor cells (OPC) are used when we analyzed specific mechanisms that can be involved in cell swelling/volume recovery in different cell types.

## Materials and methods

### Transgenic mice

Mice of postnatal age P80–120, both male and female were used in the experiments. The number of animals used in each method is stated in the method description. The experiments were performed on transgenic mice with fluorescently labeled astrocytes [line designation TgN(GFAP-EGFP), FVB background], in which the expression of EGFP was controlled by the human GFAP promoter (Nolte et al., 2001). These mice were used for comparison with other parallel studies where visualization of astrocytes is needed for morphometric experiments. The mice were either cross-bred with the Trpv4-deficient strain (on C57BL/6N background) with excised exon 12 encoding transmembrane pore domains 5 and 6 (Liedtke and Friedman, 2003), or with Aqp4-deficient mice (genetic background B6 mixed with Balb/c). Frozen embryos of the AQP4 knockout mice were obtained from Riken BRC (acc. no. CDB0758K-1^1^) and delivered by a licensed carrier to the Czechia, where breeding lines were established through embryo transfer in The Czech Centre for Phenogenomics (Vestec, Czechia). Homozygous *Trpv4*-, and *Aqp4*- deficient lines were established together with animal lines deficient in both TRPV4 and AQP4, homozygous *Aqp4-* and *Trpv4*-positive line was used as the Ctrl.

All procedures involving the use of laboratory animals were performed in accordance with the Council Directive 2010/63EU of the European Parliament and the Council of 22 September 2010, on the protection of animals used for scientific purposes and animal care guidelines approved by the Institute of Experimental Medicine, Academy of Sciences of the Czech Republic (Animal Care Committee on 7 April 2011; approval number 49/2019). All efforts were made to minimize both the suffering and the number of animals used.

### Permanent middle cerebral artery occlusion

Focal cerebral ischemia was modeled using pMCAO. The mice were anesthetized using a vaporizer (Tec-3, Cyprane Ltd., Keighley, UK) with 2% Isoflurane (Abbot, IL, USA). A part of the skin between the orbit and the external auditory meatus was cut, a 1–2 mm hole was drilled through the frontal bone and the middle cerebral artery (MCA) was occluded by electrocoagulation with bipolar tweezers (SMT, Czechia). To ensure permanent vessel occlusion, the MCA was later transected. During surgery, the body temperature was maintained at 37 ± 1°C using a heating pad (SurgiSuite, Kent Scientific, USA).

### Magnetic resonance imaging

Magnetic resonance imaging (MRI) was used to determine the extent of the post-ischemic damage after cerebral ischemia. T_2_-weighted imaging was used to quantify the volume of the ischemic lesion 1 day (D1) and 7 days (D7) after the pMCAO. Additionally, diffusion-weighted (DW) imaging was performed to obtain data on tissue diffusivity in D1. All MR experiments were performed at a 4.7 T MR scanner (Bruker BioSpec, Ettlingen, Germany) equipped with a head homemade surface coil and with a 200 mT/m gradient system (190 μs rise time). Volume analysis and diffusion results were analyzed using ImageJ software (version 1.48v, National Institutes of Health, USA).

The volume of the pMCAO-induced lesion was measured in 23 mice. The scanner uses 2D Rapid Acquisition with a Relaxation Enhancement (RARE) and a multi-spin echo sequence, allowing the acquisition of T_2_-weighted coronal and axial images. The basic sequence parameters were: Repetition Time (TR) = 3,300 ms, effective echo time (TE_eff_) = 36 ms, number of acquisitions (NA) = 1 (axial plane) and 4 (coronal plane), acquisition time = 1 min 19 s (axial plane) and 5 min 16 s (coronal plane), slice thickness = 0.6 mm, turbo factor = 8 and spatial resolution 137 μm × 137 μm, excitation and refocusing pulses: Hermite with 2.700 Hz bandwidth. The 23 mice were scanned to quantify the volume of the pMCAO-induced lesion. The lesion volumes were assessed independently by three experienced investigators. The edges of increased intensity areas were manually delineated for the axial and coronal T_2_-weighted images. The average pixel area of the two planes for each animal was calculated to determine the lesion volume. To perform the imaging, the animals were anesthetized with isoflurane (1.5% in a mixture of 40% O_2_ and 60% N_2_O) and placed in a heated mouse holder. Nine T_2_-weighted sagittal images (TE of 20 ms, TR of 2.4 s, 4 acquisitions, field of view–FOV 1.92 cm × 1.92 cm, matrix size 256 × 128, slice thickness of 0.8 mm, interslice distance 1.2 mm) were used to position the coronal slices for T_2_-weighted and DW measurements.

DW-MRI measurements were performed as previously described (Syková and Jendelová, 2005). A coronal slice was acquired using the following parameters: time interval between gradient pulses Δ = 30 ms, b-factors = 136, 329, 675, 1035, 1481, and 1825 s/mm^2^, TE = 46 ms, TR = 200 ms, field of view 1.92 cm × 1.92 cm, matrix size = 256 × 128, one 0.8 mm thick coronal slice. DW images were measured using the stimulated echo sequence. In DW measurements, the diffusion gradient direction pointed along the rostrocaudal direction. Maps of the apparent diffusion coefficient of water (ADCw) were calculated using custom-made software by a linear least square algorithm.

Data are presented as mean ± SEM. Statistical analyses of group differences were performed using a one-way ANOVA and Bonferroni’s *post hoc* test. Differences between the groups were considered statistically significant when *p* < 0.05, very significant when *p* < 0.01, and extremely significant when *p* < 0.001.

### Immunohistochemistry

For immunohistochemical analyses the experimental mice were deeply anesthetized with sodium-pentobarbital (100 mg/kg, i.p.), and transcardially perfused with 20 ml of saline with heparin (2500 IU/100 ml; Zentiva, Prague, Czechia) followed by 20 ml of 4% paraformaldehyde. Brains were dissected, post-fixed in 4% paraformaldehyde overnight, and placed stepwise in solutions with gradually increasing sucrose concentrations (10, 20, and 30%) for cryoprotection. Coronal slices (30 μm) were prepared using Hyrax C50 cryostat (Zeiss, Göttingen, Germany). The slices were incubated in a blocking solution containing 5% ChemiBLOCKER (Merck, Darmstadt, Germany) and 0.5% Triton X-100 (Merck, Darmstadt, Germany) in phosphate buffer saline (PBS) for 1 h. They were then incubated overnight at 4°C with primary antibodies diluted in a blocking solution, followed by a 2-h incubation with species-specific secondary antibodies diluted in a blocking solution at room temperature. Primary antibodies against Ca^2+^-binding adapter molecule (Iba1; diluted 1:500, Ab178846, Abcam, Cambridge, UK) and GFAP (diluted 1:800, conjugated to Cy3, C-9205, Sigma Aldrich, Merck, Darmstadt, Germany) were used. Corresponding secondary antibody (goat anti-rabbit IgG conjugated with Alexa-Fluor 594; Thermo Fisher Scientific; Waltham, MA, USA) was diluted at 1:200. After immunostaining, the slices were mounted onto microscope slides using Aqua-Poly/Mount (Polysciences Inc., Eppelheim, Germany).

### Experimental solutions

The composition of the isolation solution, artificial cerebrospinal fluid (aCSF) and the solutions modeling hypoosmotic stress (aCSF_*H*–50_ and CSF_*H*–100_), hyperkalemia (aCSF_*K+*_), and OGD are listed in Table 1. All solutions except OGD were equilibrated with 95% O_2_ and 5% CO_2_ (Carbogen, Siad, Branany, Czechia) to a final pH of 7.4, and osmolality was measured using a vapor pressure osmometer (Vapro 5520, Wescor, Logan, USA). The OGD solution was saturated with 5% O_2_, 5% CO_2_, and 90% N_2_.

**TABLE 1:** Contents of experimental solutions.

Compounds	aCSF (mM)	Isolation solution (mM)	aCSF_H–50_ (mM)	aCSF_H–100_ (mM)	aCSF_K+_ (mM)	OGD (mM)
NaCl	122	–	98	67	75	122
NMDG	–	110	–	–	–	–
KCl	3	2.5	3	3	50	3
NaHCO_3_	28	24.5	28	28	28	28
Na_2_HPO_4_	1.25	1.25	1.25	1.25	1.25	1.25
Glucose	10	20	10	10	10	–
CaCl_2_	1.5	0.5	1.5	1.5	1.5	1.5
MgCl_2_	1.3	7	1.3	1.3	1.3	1.3
Osmolality (mOsmol/kg)	∼300	∼300	∼250	∼200	∼300	∼300

aCSF, artificial cerebrospinal fluid; aCSF_H–50_, 250 mOsmol hypotonic solution; aCSF_H–100_, 200 mOsmol hypotonic solution; aCSF_K+_, 50 mM K^+^ solution; NMDG, N-methyl-D-glucamine; OGD, oxygen-glucose deprivation.

### The preparation of acute brain slices

The experimental mice were deeply anesthetized with sodium pentobarbital (100 mg/kg, i.p.; Sigma Aldrich, Germany), transcardially perfused with ice-cold (∼4°C) isolation solution and decapitated. The brains were quickly dissected, placed into the ice-cold (4°C) isolation solution, and cut transversally using a HM 650 V microtome with a vibrating blade (MICROM Int. GmbH, Waldorf, Germany). The slices were kept for 1 h in the aCSF at room temperature (23–25°C) to recover. The individual 400 μm thick coronal brain slices (from region 1.06 to 2.7 caudal from Bregma) were placed in the experimental chamber connected with an upright Zeiss microscope (Luigs and Neumann, Germany). Recordings were performed every 5 min, first at room temperature (22–25°C) and then at 32–34°C, in a chamber with aCSF enriched with 0.1 mM tetramethylammonium ion (TMA^+^) at a flow rate of 4 ml/min, which was a continuously bubbled with 95% O_2_ and 5% CO_2_.

### Measurements of the extracellular space diffusion parameters

The ECS diffusion parameters were measured in acute brain slices under the exposure to four different models of brain pathology, resulting in osmotic disbalance and cell swelling—aCSF_*H*–50_, aCSF_*H*–100_, aCSF_*K+*_, and OGD. The data were averaged from measurements obtained from 48 Ctrl, 39 AQP4^–/–^/TRPV4^–/–^, 43 AQP4^–/–^, and 46 TRPV4^–/–^ mice. Data from 22 Ctrl were adopted from our previous study (Chmelova et al., 2019), and pooled with additional experiments in 26 mice that confirmed the previous results. Similarly, new additional experiments extended the number of AQP4^–/–^ and TRPV4^–/–^ mice in this study by 23 and 30 animals, respectively. The ECS volume fraction α (α = ECS volume/total tissue volume), tortuosity λ (λ^2^ = free diffusion coefficient/apparent diffusion coefficient), and non-specific uptake (k′ [s^–1^]), were determined by the RTI method previously described by Nicholson and Phillips (1981). Briefly, the ECS marker TMA^+^ (MW 74.1 Da) is administered into the tissue through the iontophoretic microelectrode, and its concentration is measured using a double-barreled ion-selective microelectrode (ISM) (Syková, 1992). The double-barreled ISM consists of a reference barrel containing 150 mM NaCl and an ion-sensitive barrel filled with ion-exchanger IE 190 (WPI, Inc., Sarasota, USA)^2^ at the tip, and backfilled with 100 mM solution of TMA^+^. The electrodes were calibrated before each experiment in a series of solutions with increasing TMA^+^ concentrations of 0.25, 0.5, 1, 2, 4, 8, and 16 mM in a background of 150 mM NaCl and 3 mM KCl. The TMA^+^ signal was recorded by a hand-manufactured differential amplifier, which amplifies the voltage difference by subtracting the background signal common to both barrels, then digitizes and stores them on a personal computer. Obtained voltages were fitted to the Nikolsky equation to acquire the slope and the interference of each ISM. The iontophoretic microelectrode was filled with 100 mM TMA^+^ and was glued to individual ISM with a tip separation of 80–100 μm (Supplementary Figure 1). Prior to the tissue measurements, the ISMs were calibrated in 0.3% agar gel (Sigma Aldrich, Germany), dissolved in a solution of 150 mM NaCl, 3 mM KCl, and 1 mM TMA^+^, in which α = 1, λ = 1, and *k*′ = 0 s^–1^ (free diffusion values). During calibration, a 20 nA bias current was continuously applied from a constant current, high impedance source (Single Channel Iontophoresis Generator ION-100; Dagan Corporation, Minneapolis, Minnesota, USA), to maintain a constant electrode transport number. A current step of 200 nA and 24 s duration generated a diffusion curve (stimulator Master 8, A.M.P.I, Jerusalem, Israel). The diffusion curves obtained in agar were analyzed by a non-linear curve fitting simplex algorithm, operating on a modified diffusion equation using the VOLTORO program (kindly provided by C. Nicholson, New York University School of Medicine, USA, unpublished data), to acquire the values of the free diffusion coefficient of TMA^+^ (D) and the electrode transport number (*n*). Knowing the *n* and D values, the parameters α, λ, and k′ could be determined from the slice. To decrease the potential toxic effect of TMA^+^ on the tissue, the background of TMA^+^ in the solution was lower (0.1 mM) than in agar (1 mM), where a relatively high TMA^+^ concentration is used to obtain a stable value of the transport number. To verify that all slices are viable and non-ischemic, the diffusion experiments *in situ* were first performed at aCSF at room temperature (24°C), and the obtained values were compared to our database of the extracellular diffusion parameter values from the previous studies during normoxia and anoxia; if the value of α was lower than 0.10 and, concomitantly, λ was higher than 1.80, the slice was considered to be anoxic. Previous studies (Shibasaki et al., 2015; Salman et al., 2017) demonstrated that TRPV4 activity is inhibited at room temperature. To activate the TRPV4 channels and maintain viable slices we raised the temperature of the bath solution to 32–34°C, which represents a compromise value, that activates the TRPV4 channels but does not harm the slices. After stabilization of the ECS diffusion parameter values, we began the 20-min application of aCSF_*H*–50_, aCSF_*H*–100_, OGD, or aCSF_*K+*_ solutions followed by a 20-min washout in aCSF. The diffusion curves were captured and analyzed every 5 min in the brain cortex at a depth of 200 μm.

Data are expressed as mean ± SEM, N represents the number of animals in the group, and *n* represents the number of slices. The statistical analyses of the differences in ECS diffusion parameters among the experimental groups, and of the ECS diffusion parameter, changed within the experimental groups during applications, were performed using two-way ANOVA with Tukey’s post-test. The differences were considered significant when the value of *p* < 0.05. If not stated otherwise, all datasets conform to the assumption of ANOVA model and Tukey’s test, i.e., normal distribution and variance homogeneity verified by a D’Agostino-Pearson omnibus K2 test (D’Agostino, 1986).

### Measurements of extracellular K^+^ concentration

The experiments were performed on 15 Ctrl, 10 AQP4^–/–^, 9 TRPV4^–/–^, and 13 AQP4^–/–^/TRPV4^–/–^ animals. The extracellular potassium concentration ([K^+^]_*o*_) was measured by double-barreled K^+^-sensitive microelectrodes, as previously described in detail (Svoboda and Sykova, 1991). Briefly, the tip of the K^+^-selective barrel of the microelectrode was filled with the liquid ion-exchanger IE 190 [WPI, Inc., Sarasota, USA (see text footnote 2)] and back-filled with 100 mM KCl, whereas the reference barrel contained 150 mM NaCl. Electrodes were calibrated in a sequence of solutions containing 2, 5, 8, 20, 50, and 80 mM KCl, with a background of either 148, 145, 142, 130, 100, or 70 mM NaCl, to keep the ionic strength of the solution constant. The data were fitted to the Nikolsky equation to determine the electrode slope. The measurements were performed in the cortex *in situ* and to activate the TRPV4 channels and maintain viable slices, we raised the temperature of the bath solution to 33°C. After the stabilization of the [K^+^]_*o*_ values, we began the 20-min application of OGD solution followed by a 20-min washout in aCSF. The voltage was captured every 1 min. Based on the electrode characteristics, the measured voltage was converted to extracellular concentrations by implementing modified Nernst equation. Data are presented as the mean ± SEM. Statistical analyses were performed by two-way ANOVA with Tukey’s post-test. Differences between the groups were considered statistically significant when *p* < 0.05, very significant when *p* < 0.01, and extremely significant when *p* < 0.001.

## Results

In this study, we used MRI to quantify the impact of pMCAO on the size of ischemic brain damage in mice lacking AQP4, TRPV4, or AQP4/TRPV4; the observed results were verified by immunohistochemistry. To elucidate function of AQP4 and TRPV4 in different models of acute edema, we employed the RTI method to determine changes of the ECS diffusion parameters induced by hypoosmotic stress, hyperkalemia or OGD.

### The impact of AQP4 and TRPV4 deletion on the volume of the ischemic lesion

**FIGURE 1:**
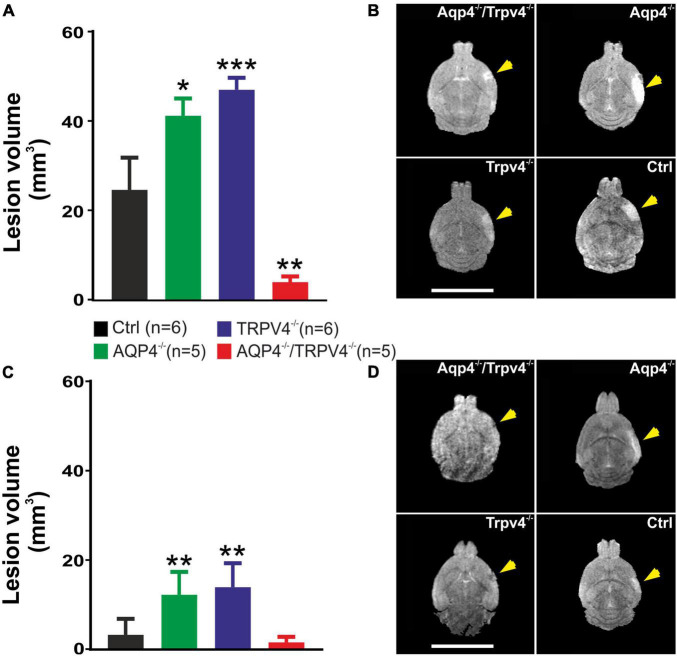
Quantification of the ischemic lesion volume using T_2_-weighted magnetic resonance imaging of the mouse brain 1 day (D1) and 7 days (D7) after pMCAO. **(A)** Bar graph showing an average volume of the ischemic lesion in the AQP4^–/–^, TRPV4^–/–^, and AQP4^–/–^/TRPV4^–/–^ mice compared to the Ctrl at D1 after pMCAO. Note that mice lacking AQP4 or TRPV4 channels showed significantly higher lesion volume, whereas the volume of the lesions in the mice lacking both channels was significantly reduced when compared to the single knockouts. **(B)** Representative T_2_-weighted images of the brains of the Ctrl, AQP4^–/–^, TRPV4^–/–^, and AQP4^–/–^/TRPV4^–/–^ mice at D1 after pMCAO. The lesion is visible as a mild hyperintense area in the left hemisphere (yellow arrowheads). **(C)** Bar graph showing an average volume of the ischemic lesion in the AQP4^–/–^, TRPV4^–/–^, and AQP4^–/–^/TRPV4^–/–^ mice compared to the Ctrl at D7 after pMCAO. Note that at D7 after pMCAO the lesion volume remains larger in the AQP4^–/–^ as well as TRPV4^–/–^ mice compared to the Ctrl, but in the AQP4^–/–^/TRPV4^–/–^, the lesion size is comparable to Ctrl. **(D)** Representative T_2_-weighted images of the brains of the Ctrl, AQP4^–/–^, TRPV4^–/–^, and AQP4^–/–^/TRPV4^–/–^ mice at D7 after pMCAO. The lesion is visible as a mild hyperintense area in the left hemisphere (yellow arrowheads). Data are presented as mean + SEM. Asterisks indicate significant differences between the AQP4^–/–^/TRPV4^–/–^ mice and the Ctrl (*p < 0.05, **p < 0.01, ***p < 0.001). Ctrl, control; AQP4^–/–^, AQP4-deficient mice; TRPV4^–/–^, TRPV4-deficient mice; AQP4^–/–^/TRPV4^–/–^, AQP4- and TRPV4-deficient mice; pMCAO, permanent middle cerebral artery occlusion.

We quantified here the volume of the ischemic lesion in AQP4^–/–^ and TRPV4^–/–^ mice, and in mice with the simultaneous deletion of both channels (AQP4^–/–^/TRPV4^–/–^) employing T2-weighted MRI at D1 and D7 following pMCAO (Figure 1), to assess their role in edema formation and the post-ischemic outcome of the brain tissue. The results were compared to those obtained in the Ctrl mice. Our experiments showed that the AQP4^–/–^ as well as the TRPV4^–/–^ mice develop about a twice larger lesion volume 1D after pMCAO when compared to the Ctrl. Similarly, the volume of the lesion at D7 after pMCAO was significantly enlarged in both the TRPV4^–/–^ as well as AQP4^–/–^ mice, when compared to the Ctrl. The decrease in the lesion volume between D1 and D7 was comparable in both the single knockouts, and did not significantly differ from that in the Ctrl mice (Table 2).

**TABLE 2:** Absolute values of the lesion volume (mm^3^) determined by T_2_-weighted magnetic resonance imaging of the mouse brain 1 day (D1) and 7 days (D7) after pMCAO.

	D1	D7	% of decrease	N
Ctrl	24.85 ± 7.29	3.32 ± 1.50	87.52 ± 2.31	6
Aqp4^–/–^	41.51 ± 3.90	12.35 ± 2.30	70.61 ± 4.57	5
Trpv4^–/–^	47.37 ± 2.70	14.08 ± 2.42	71.11 ± 13.47	5
Aqp4^–/–^/ Trpv4^–/–^	4.15 ± 1.29	1.64 ± 0.56	54.05 ± 14.79	5

Ctrl, control; Aqp4^–/–^, AQP4-deficient mice; Trpv4^–/–^, TRPV4-deficient mice; Aqp4^–/–^/Trpv4^–/–^, AQP4- and TRPV4-deficient mice; N represents the number of animals in the group.

Interestingly, quantification of a lesion volume in the AQP4^–/–^/TRPV4^–/–^ mice brought quite the opposite results, revealing a markedly smaller lesion volume at D1 when compared to both the single knockouts (Figure 1 and Table 2). The lesser impact of ischemic injury on the AQP4^–/–^/TRPV4^–/–^ mice at D1 was also confirmed by the DW-MRI. While in the AQP4^–/–^/TRPV4^–/–^ mice, the ADC_*W*_ values on the ipsilateral side (609 ± 16 μm^2^s^–1^) did not significantly differ from the contralateral side (601 ± 13 μm^2^s^–1^), in the Ctrl mice, the ADC_*W*_ on the ipsilateral side was significantly lower than on the contralateral side (481 ± 17 μm^2^s^–1^ and 665 ± 18 μm^2^s^–1^, respectively; *p* < 0.001). Compared to the Ctrl, the values of the lesion volume in the AQP4^–/–^/TRPV4^–/–^ at D7 were not significantly different, (Figure 1 and Table 2). However, they were still significantly smaller when compared to both the single knockouts. The decrease in the lesion volume in AQP4^–/–^/TRPV4^–/–^ mice between D1 and D7 was comparable to that observed in both the single knockouts and Ctrl mice.

**FIGURE 2:**
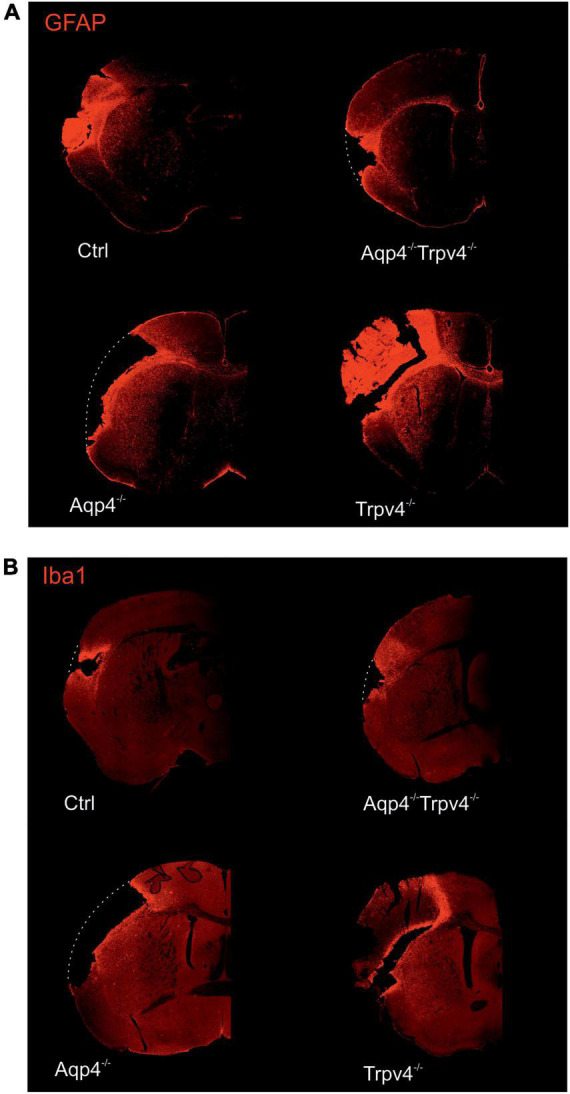
Immunohistochemical staining of the brains 7 days (D7) after pMCAO. Staining with antibodies against GFAP **(A)** and Iba1 **(B)** showed that the extent of the damage in Aqp4^–/–^/Trpv4^–/–^ mice is comparable to the Ctrl. Conversely, significantly greater damage was observed in the Aqp4^–/–^/or Trpv4^–/–^ compared to the Ctrl and the Aqp4^–/–^/Trpv4^–/–^ mice. The white dotted lines show the original size of the tissue slices in the places, where due to the extent of the ischemic damage some parts of the slices fell out. Details of the pictures showing GFAP or Iba1 expression are shown at right in white frames. Ctrl, control; AQP4^–/–^, AQP4-deficient mice; TRPV4^–/–^, TRPV4-deficient mice; AQP4^–/–^/TRPV4^–/–^, AQP4- and TRPV4-deficient mice; GFAP, glial fibrillary acidic protein; Iba1, ionized Ca^2+^-binding adapter molecule.

The differences in the extent of the tissue damage observed between the single knockouts, AQP4^–/–^/TRPV4^–/–^ and Ctrl, were confirmed using immunohistochemical staining performed at D7 after pMCAO (Figure 2). We used antibodies against GFAP and Iba1 to visualize reactive astrocytes and microglia, respectively. Consistent with the MRI results, we observed comparable damage in the AQP4^–/–^/TRPV4^–/–^ as in the Ctrl mice, and significantly larger damage in the AQP4^–/–^ or TRPV4^–/–^ mice (Figure 2).

In summary, similarly to the deletion of TRPV4 (Pivonkova et al., 2018), the deletion of AQP4 also worsens the impact of ischemic injury in the acute (D1), as well as chronic, phase (D7) following pMCAO. On the contrary, the simultaneous deletion of AQP4 and TRPV4 channels appears to be protective in the acute phase, while in the chronic phase the outcomes of ischemic injury in the Ctrl and double knockouts are comparable.

### The effect of AQP4 and TRPV4 deletion on the diffusion parameters of the extracellular space in response to pathological stimuli

To assess how the post-ischemic alterations on the whole brain level (MRI data) correspond with the changes on the tissue and cellular level, we employed the RTI method for measurements of the ECS diffusion parameters: α, λ, and k′. At the beginning of each experiment in acute brain slices, the control values of α, λ, and k′ were obtained from the cortex bathed in 22–24°C aCSF and averaged. When these values indicated that the slice is viable (see section “Materials and methods”), the temperature of aCSF was increased to 33°C to activate the TRPV4 channels. The resting values of α, λ, or k′ in the AQP4^–/–^/TRPV4^–/–^ mice in either 22–24°C or 33°C matched the values in the Ctrl (Supplementary Table 1). Our previously published data (Chmelova et al., 2019), confirmed also by this study, showed a significantly higher resting value of α in the mice deficient for AQP4 in comparison with the Ctrl, which persisted on the lower level of significance when the temperature was increased. On the other hand, the resting α values from TRPV4^–/–^ and the Ctrl mice did not differ significantly. We did not detect any significant alterations among the experimental groups in the resting values of λ or k′ (Supplementary Table 1).

#### Oxygen-glucose deprivation

**FIGURE 3:**
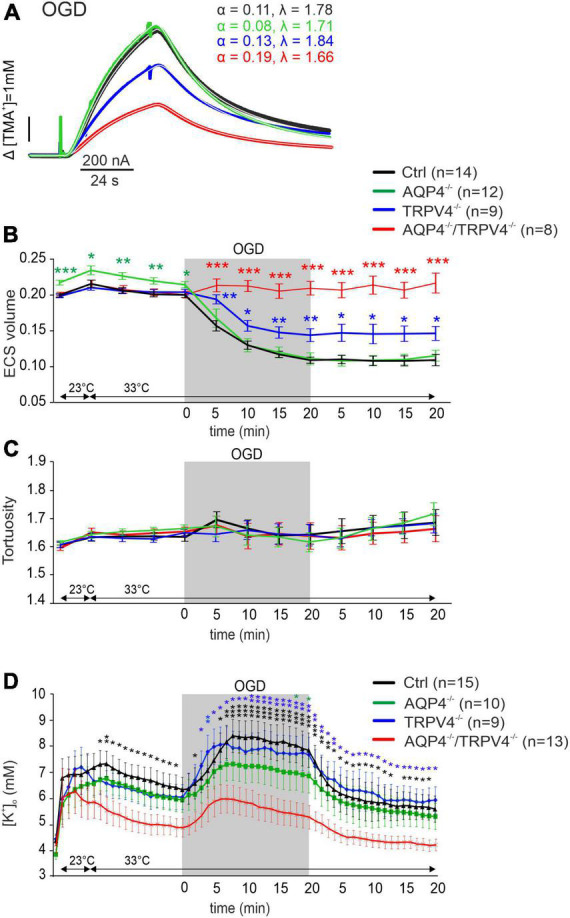
The effect of oxygen-glucose deprivation (OGD) on the ECS diffusion parameters and extracellular potassium concentration [K^+^]_o_ *in situ*. **(A)** Representative diffusion curves with the corresponding values of the ECS volume fraction (α) and tortuosity (λ) recorded in the Ctrl, AQP4^–/–^, TRPV4^–/–^, and AQP4^–/–^/TRPV4^–/–^ mice. Averaged data of α **(B)** and λ **(C)** measured in the Ctrl, AQP4^–/–^, TRPV4^–/–^, and AQP4^–/–^/TRPV4^–/–^ mice at resting conditions (22–24°C), 33°C and at 5-min intervals during and after (washout) OGD. Asterisks indicate significant differences between Ctrl and AQP4^–/–^, TRPV4^–/–^, or AQP4^–/–^/TRPV4^–/–^ mice (*p < 0.05, **p < 0.01, ***p < 0.001). **(D)** Values of [K^+^]_o_ recorded in 1-min intervals in the Ctrl, AQP4^–/–^, TRPV4^–/–^, and AQP4^–/–^/TRPV4^–/–^ mice. Data are presented as mean ± SEM. Asterisks indicate significant differences between AQP4^–/–^/TRPV4^–/–^ mice and AQP4^–/–^, TRPV4^–/–^, or Ctrl (*p < 0.05, **p < 0.01, ***p < 0.001). Ctrl, control; AQP4^–/–^, AQP4-deficient mice; TRPV4^–/–^, TRPV4-deficient mice; AQP4^–/–^/TRPV4^–/–^, AQP4- and TRPV4-deficient mice; OGD, oxygen-glucose deprivation; *n*, number of slices; ECS, extracellular space.

Oxygen-glucose deprivation is frequently used as a model of stroke *in situ* because of its similarity to models of cerebral ischemia *in vivo* (Sommer, 2017). To study the developing tissue changes evoked by an acute cell swelling, we recorded the values of ECS diffusion parameters every 5 min during a 20-min application of OGD, as well as during the subsequent 20-min washout (Figure 3). During OGD, we detected a significant decrease in α (*p* < 0.001) in the Ctrl mice, in comparison with its resting values but no changes in λ or k′ (Figure 3 and Supplementary Table 1). Surprisingly, in AQP4^–/–^/TRPV4^–/–^ the α, λ, and k′ remained unaffected by OGD application throughout the whole time-course of the experiment (*p* > 0.99, *p* > 0.98, and *p* > 0.86, respectively; evaluated in the 20th minute of application; Figure 3 and Supplementary Table 1). It should be noted that we already described in our previous publication that a decrease in α evoked by OGD in AQP4^–/–^ mice is comparable to the Ctrl; however a decrease in the TRPV4^–/–^ mice was significantly smaller than in the Ctrl (Chmelova et al., 2019). The α values in the Ctrl as well as in both single knockout strains remained decreased even during 20 min of washout (Chmelova et al., 2019).

Since ischemia is accompanied by a profound increase in [K^+^]_*o*_, we also estimated extracellular K^+^ concentrations evoked by OGD, with the expectation that they may differ in the Ctrl and AQP4^–/–^/TRPV4^–/–^ mice. The value of [K^+^]_*o*_ at room temperature was similar in both the Ctrl and AQP4^–/–^/TRPV4^–/–^ mice (4.5 ± 0.4 mM and 4.2 ± 0.3 mM). Significant differences in [K^+^]_*o*_ between the Ctrl and AQP4^–/–^/TRPV4^–/–^ appeared at the elevation of bath temperature to 33°C, and were pronounced during OGD with a maximum increase in the Ctrl mice to 8.4 ± 0.5 mM and in the AQP4^–/–^/TRPV4^–/–^ animals to 5.9 ± 0.5 mM. These differences also persisted during washout (Figure 3D). To improve understanding of such low [K^+^]_*o*_ in the cortex of the AQP4^–/–^/TRPV4^–/–^ mice, we also estimated [K^+^]_*o*_ in the cortex of the single AQP4 or TRPV4 knockouts during OGD. There were no significant differences in [K^+^]_*o*_ between the single knockouts and Ctrl during OGD, with maximum values of 7.3 ± 1.0 mM in AQP4^–/–^ mice, and 8.1 ± 0.6 mM in TRPV4^–/–^ mice (Figure 3).

#### Hypoosmotic stress

**FIGURE 4:**
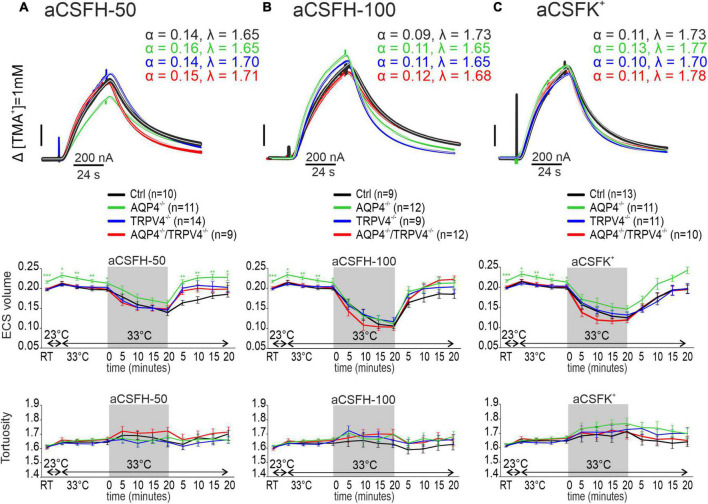
The effect of hypoosmotic stress **(A,B)** and hyperkalemia **(C)** on the ECS diffusion parameters *in situ*. Representative diffusion curves with the corresponding values of the ECS volume fraction (α) and tortuosity (λ) recorded in the Ctrl, AQP4^–/–^, TRPV4^–/–^, and AQP4^–/–^/TRPV4^–/–^ mice (upper panels). Averaged data of α (middle panels) and λ (bottom panels) measured in the Ctrl, AQP4^–/–^, TRPV4^–/–^, and AQP4^–/–^/TRPV4^–/–^ mice at resting conditions (22–24°C), 33°C and at 5-min intervals during and after (washout) applications of aCSF_H–50_, aCSF_H–100_, and aCSF_K+_ solutions. Data are expressed as mean ± SEM. No significant differences were detected between the AQP4^–/–^/TRPV4^–/–^ and control animals. Ctrl, control; AQP4^–/–^, AQP4-deficient mice; TRPV4^–/–^, TRPV4-deficient mice; AQP4^–/–^/TRPV4^–/–^, AQP4- and TRPV4-deficient mice; aCSF_H–50_, 250 mOsmol hypotonic solution; aCSF_H–100_, 200 mOsmol hypotonic solution; aCSF_K+_, 50 mM K^+^ solution.

Hypoosmotic stress was used as another model of the conditions inducing cytotoxic edema. Two different solutions modeling either mild (aCSF_*H*–50_) or severe (aCSF_*H*–100_) hypoosmotic stress were used (Supplementary Table 1). In all the experimental groups, α was significantly reduced by the application of both aCSF_*H*–50_ and aCSF_*H*–100_ (*p* < 0.001 in all groups) but no significant changes between the Ctrl and AQP4^–/–^/TRPV4^–/–^ were observed. Hypoosmotic conditions did not induce any significant changes in λ or k′ (Figures 4A,B and Supplementary Table 1). In our previous publications, we already demonstrated that neither AQP4 nor TRPV4 deletion alone had an impact on the maximal decrease of α values during hypoosmotic stress (Pivonkova et al., 2018; Chmelova et al., 2019). During washout, the α gradually returned to the resting values in slices from the Ctrl and AQP4^–/–^/TRPV4^–/–^ mice. Similar behavior during the washout period was also observed in the TRPV4^–/–^ and AQP4^–/–^ (Chmelova et al., 2019).

#### Hyperkalemic conditions

As the last ischemia mimicking model we employed extracellular hyperkalemia, induced by the application of 50 mM potassium solution (aCSF_*K+*_, Supplementary Table 1). Similarly, as in hypoosmotic stress, α values significantly decreased during the application of aCSF_*K+*_ solution in both the experimental groups (*p* < 0.001), but no differences were observed between the AQP4^–/–^/TRPV4^–/–^ mice and Ctrl. Hyperkalemic conditions did also not induce any significant changes in λ or k′ in any of the experimental groups (Figure 4C and Supplementary Table 1). During washout, α gradually returned to the resting values.

We conclude that the simultaneous deletion of the AQP4 and TRPV4 channels leads to reduced swelling of the cellular components of the brain tissue caused by OGD, which is manifested as having no reduction in extracellular space volume. Moreover, we showed that [K^+^]_o_ concentration reaches lower values in the AQP4^–/–^/TRPV4^–/–^ mice than in the Ctrl during OGD. These results are consistent with the data we obtained from MRI, which show that the extent of ischemic damage is significantly lower in the AQP4^–/–^/TRPV4^–/–^ mice than in the Ctrl at D1 after pMCAO. Therefore, our findings support the hypothesis that the AQP4 and TRPV4 cooperate in OGD-induced cell swelling, and are responsible for the development of cerebral edema and ischemic brain damage.

## Discussion

In this study, we demonstrate that both AQP4 and TRPV4 channels, particularly their interaction, play an important role in edema formation after ischemia *in vivo* and under ischemia-like conditions such as OGD *in situ*.

### The deteriorating effect of AQP4 or TRPV4 deletion on the size of ischemic lesion

The quantification of MRI images revealed that mice lacking either AQP4 or TRPV4 channels display significantly enlarged ischemic lesions after pMCAO, when compared to mice carrying both channels. In addition, our immunohistochemical analyses employing antibodies against GFAP and Iba1 supported MRI data, by revealing enlarged ischemic lesions in both the single knockouts, and the presence of reactive astrocytes and microglia within the damaged hemisphere. Our single knockout data suggest a protective role of AQP4 and TRPV4 during the development of brain edema, especially in the acute phase of ischemic injury (D1). Furthermore, the deletion of either AQP4 or TRPV4 has a strong impact on the final extent of ischemic damage as seen in D7. A similar finding was demonstrated by Zheng and co-authors and others (Shi et al., 2012; Zeng et al., 2012), who described that AQP4 knockout aggravates ischemia/reperfusion injury in mice. In contrast, our data do not fully correspond with those showing a reduced lesion size in the TRPV4^–/–^ mice (Tanaka et al., 2020), and those reporting reduced brain edema and infarct volume in AQP4-deficient mice (Yao et al., 2015). It must be noted that in these studies transient MCAO (tMCAO) was employed, which results in more severe ischemic injury when compared to our model of pMCAO (Kriska et al., 2021). Such discrepancies may dwell from employing different types of knockout mice as well as the type of MCAO.

In general, there are controversies in the literature concerning the protective or detrimental effect of AQP4 deletion in different pathologies where brain edema occurs. Katada et al. (2014) showed reduced brain swelling after bilateral transient carotid artery occlusion, and Manley et al. (2000) demonstrated reduced brain swelling after water intoxication. An improved long-term outcome, including decreased mortality and increased motor recovery associated with decreased lesion volume, neuronal cell death, and neuroinflammation, was also described in the AQP4^–/–^ mice after transient cerebral ischemia (Hirt et al., 2017). However, the opposite effect of AQP4 deletion was shown during the development of brain edema associated with bacterial infection, where the deletion of AQP4 led to an increase in the brain water content as well as increased brain swelling (Bloch et al., 2005). Such discrepancies in the effect of AQP4 deletion on brain swelling are very likely caused by the different mechanisms of tissue swelling employed in each model, and by the contribution of vasogenic and cytotoxic types of brain edema. Moreover, the contribution of AQP4 channels to cell swelling can vary in different models of cytotoxic edema. For example, in our previous studies in α-syntrophin (α-syn) knockout mice with reduced AQP4 levels in perivascular and subpial membranes, we observed reduced edema formation as well as slower volume recovery under severe pathological conditions or in states associated with elevated K^+^ (Dmytrenko et al., 2013; Anderova et al., 2014). Similarly, AQP4 deletion slowed down the development of brain ADC_W_ changes in terminal ischemia *in vivo*, but did not affect changes in diffusion parameters during hypoosmotic stress or OGD in tissue slices (Chmelova et al., 2019). According to these studies, it seems that the protective mechanism of AQP4 deletion includes the prevention of water accumulation in the tissue that reduces the extent of brain edema.

A number of studies have shown that AQP4 co-localizes with various channels/transporters and its knockout significantly deteriorates the functions of Kir4.1, type 2 chloride channels (ClC2), VRAC and glutamate uptake (Amiry-Moghaddam et al., 2004; Benfenati et al., 2007; Zeng et al., 2007; McCoy et al., 2010; Reed and Blazer-Yost, 2022) involved in ion, volume and glutamate homeostasis. Thus, the resulting detrimental or beneficiary effect of AQP4 deletion might depend on the prevailing activity of different transport mechanisms that may vary in time. Moreover, it has been shown that AQP4 channels play an inhibitory role in post-ischemic inflammation caused by microglia (Shi et al., 2012). Aggravated inflammation in AQP4^–/–^ may thus significantly enlarge post-ischemic lesion especially in the late phase of ischemia (D7).

The role of TRPV4 in the brain edema formation was reported in the TRPV4^–/–^ animals, where TRPV4 deficiency led to the reduction of cell swelling and amelioration of peri-infarct depolarization (Jie et al., 2015; Rakers et al., 2017; Hoshi et al., 2018), as well as to the decrease in ischemia-induced lesion volume and milder neurological symptoms (Tanaka et al., 2020). Similarly, a TRPV4 blockade by antagonists in a model of intracerebral hemorrhage preserved the BBB and attenuated neurological deficits (Zhao et al., 2018). The neuroprotective effect of TRPV4 inhibition was also confirmed in a tMCAO model, where improved microcirculation and BBB function were observed in the TRPV4^–/–^ mice compared to the controls (Tanaka et al., 2020). On the contrary, our recent study showed that TRPV4 deletion aggravated the extent of brain edema as was evaluated 1 and 7 days after pMCAO (Pivonkova et al., 2018). This finding was confirmed in the current study, and similar aggravation after pMCAO was detected in the AQP4^–/–^ mice. In a recent work, Rosenkranz et al. (2020) observed no protection in mice lacking TRPV4 in various pathological models, such as multiple sclerosis, experimental autoimmune encephalomyelitis and transient MCAO. Of note, they found no differences in a size of the lesion (Rosenkranz et al., 2020).

It has been shown that increased TRPV4 activity affects and changes the expression/function of several membrane proteins such as BKCa channels, NMDA receptors, α-amino-3-hydroxy-5-methyl-4-isoxazole-propionate receptors (AMPARs), inositol 1,4,5-trisphosphate receptors (IP3Rs), ryanodine receptors (RyRs), AQP4, and other potential cooperative receptors in the brain (Liu et al., 2020). As TRPV4 is expressed on the plasma membrane, it may interact with other channels and play a crucial role in the nervous system activity. Since TRPV4 co-localizes with AQP4 (Benfenati et al., 2007; Jo et al., 2015), TRPV4 knockout may also affect the function of the AQP4-co-expressing channels (Mola et al., 2021). Under some pathological conditions such as ischemia, TRPV4 channels are upregulated (Butenko et al., 2012) and sensitized *via* cellular signaling pathways, and this can cause additional tissue damage.

Unlike AQP4, which is expressed predominantly in astrocytes (Saadoun et al., 2005; Verkman, 2013), TRPV4 channels are expressed more widely. In neurons, TRPV4 is involved in the modulation of neuronal excitability (Shibasaki et al., 2015). In astrocytes they are abundant at astrocytic endfeet processes, which wrap around blood vessels and thus might play a key role in brain volume homeostasis (Benfenati et al., 2007, 2011). Recent research discovered TRPV4 channels on oligodendrocyte precursor cells (OPC), where their activation promotes OPC proliferation (Ohashi et al., 2018). It has also been shown that TRPV4 regulates the activity of microglia (Konno et al., 2012) and plays a role in the regulation of cerebral perfusion through its effect on smooth muscles (Diaz-Otero et al., 2019). Due to its broad expression in various cell types, TRPV4 may be activated during ischemia by multiple stimuli, and involved in various functions. For example, TRPV4-mediated Ca^2+^ influx in astrocytic endfeet contributes to the response to neuronal activation, enhances the accompanying vasodilation and contributes to neurovascular coupling (Dunn et al., 2013). Activated endothelial and smooth muscle TRPV4 channels can mediate the activation of large-conductance calcium-activated potassium channels (BKCa) leading to cerebral arterial dilation, which can improve hypoperfusion in the infarcted area (Han et al., 2018). On the other hand, blocking TRPV4 channels by their antagonist HC-067047 decreases cerebral ischemic injury in mice through the prevention of N-methyl-D-aspartate (NMDA) receptors-induced glutamate excitotoxicity (Li et al., 2013). It has been shown that heat-sensitive TRPV4 and cold-sensitive TRPM8 ion channels are involved in microglial activity regulation (Chakraborty and Goswami, 2022). Redmon et al. (2021) demonstrated that the TRPV4 channel may represent a primary retinal microglial sensor of osmo-challenges under physiological and pathological conditions and suggested that TRPV4 inhibition might be a useful strategy to suppress microglial overactivation in the swollen and edematous CNS.

Thus, changes in TRPV4 expression and its activity as possible treatments for cerebral ischemia remain unclear. It appears that several channels/transporters can serve as TRPV4 effector proteins and a dual effect of TRPV4 blockage (especially in the acute or delayed phase of ischemia) may be caused by a “switch” between various effectors. Furthermore, we must consider that reduced transmembrane water transport, due to AQP4 deficiency, may slow down the development of cytotoxic edema, but it also slows down the volume regulation during reperfusion. Thus, the early “beneficial” effect may become “detrimental” due to prolonged cell swelling and delayed normalization of volume and ion conditions in the tissue.

### The simultaneous deletion of AQP4 and TRPV4 strongly attenuates brain edema formation in the acute post-ischemic phase

Interestingly, the simultaneous deletion of both channels had quite the opposing effect than in the single knockouts, resulting in a significant reduction of the size of ischemic lesion 1 day after pMCAO and less affected ADC_W_ in the AQP4^–/–^/TRPV4^–/–^ mice, when compared to the Ctrl. However, after 7 days the lesions in the AQP4^–/–^/TRPV4^–/–^ mice were comparable to those observed in the Ctrl mice. Of note, employing several behavioral tests (Rotarod, Cylinder and Tape removal test) one and 7 days (resp. 8 days) after pMCAO revealed that both groups (Ctrl and AQP4^–/–^/TRPV4^–/–^) performed similarly (unpublished data). The question still remains as to whether employed behavioral tests have adequate sensitivity to disclose even subtle differences between the Ctrl and double knockout mice, following pMCAO (Ruan and Yao, 2020).

This response of the AQP4^–/–^/TRPV4^–/–^ mice to pMCAO indicates an interplay between these two channels, which was previously suggested in the experiments using cultured glia (Benfenati et al., 2011; Jo et al., 2015; Redmon et al., 2021) or in meningiomas (Faropoulos et al., 2021), where AQP4-mediated water fluxes promote the activation of TRPV4, whereas Ca^2+^ entry through TRPV4 channels reciprocally modulates volume regulation, swelling, and Aqp4 gene expression (Jo et al., 2015). This interplay is especially prominent during the acute phase of ischemia, because after 7 days the damage detected in the AQP4^–/–^/TRPV4^–/–^ mice was comparable to that observed in the Ctrl. Therefore, we conclude that the deletion of both AQP4 and TRPV4 channels may have a protective role during the acute stages of ischemic injury (D1), mostly during the development of brain edema.

The mechanism underlying the interaction between TRPV4 and AQP4 is still not well understood. It is well established that TRPV4 and AQP4 are co-expressed in the plasma membranes of astrocytes and jointly trigger RVD (Benfenati et al., 2011). Moreover, a recent study provided evidence that high expression of TRPV4 and AQP4 induces cell swelling in brain slices under ischemic conditions, while TRPV4 and AQP4 deficits decelerate extracellular space shrinkage (Chmelova et al., 2019). After rapid entry of water through AQP4, swelling-induced Ca^2+^ influx *via* TRPV4 channels and Ca^2+^-induced Ca^2+^ release from the endoplasmic reticulum is modulated by IP3R2 receptors in different astrocytic territories (Eilert-Olsen et al., 2019). The influx of Ca^2+^ through TRPV4 channels quickly increases the osmotic gradient, resulting in the movement of water through AQP4. However, another study reported that for osmosensation, TRPV4 activation is independent of osmolality-associated AQP4 permeability (Wang and Parpura, 2018). Therefore, further studies of the interaction between TRPV4 and AQP4 would be beneficial.

### The simultaneous deletion of AQP4 and TRPV4 block changes of the extracellular space diffusion parameters during oxygen-glucose deprivation

In this study, we also employed the RTI method *in situ* models of acute cell swelling, which allows the detection of changes in the ECS diffusion parameters that indirectly reflect swelling and/or cell volume regulation, as the increase of the cellular volume is accompanied by a compensatory shrinkage of the ECS volume and vice versa. Volume fraction α reflects volume changes in all cellular elements of the tissue, which may swell and regulate their volume in a different way than astrocytes (Andrew et al., 2007; Caspi et al., 2009; Murphy et al., 2017). In our previous study, we demonstrated that the lack of either AQP4 or TRPV4 slows down the development of cytotoxic edema in terminal ischemia and TRPV4 deletion attenuates the ECS volume decrease induced by OGD treatment *in situ*. Nevertheless, AQP4 deletion had no impact on tissue diffusivity during OGD (Chmelova et al., 2019). We confirmed these results here in both the single knockout strains and, most importantly, we disclosed that the simultaneous deletion of AQP4 and TRPV4 channels completely blocks the tissue response to OGD treatment in acute brain slices.

Oxygen-glucose deprivation treatment became a widely used *in situ* model of ischemia, as several studies reported that the perfusion of the slices with low oxygen and glucose-free solution mimics fairly accurately the ischemic conditions occurring *in vivo* (Richard et al., 2010; Holloway and Gavins, 2016). However, in contrast to *in vivo* conditions, *in situ* perfusion of the slices with the bath solution allows the removal of the toxic metabolites, which could alleviate tissue damage. During OGD treatment, we observed a smaller α decrease in TRPV4^–/–^ than in the Ctrl or AQP4^–/–^ mice, with no observed recovery of α within 20 min of washout in all three groups (Supplementary Table 2; Chmelova et al., 2019). Similar findings were shown in the study of Pivonkova et al. (2018), who employed 3D morphometry to quantify single astrocyte volume changes. These results indicate that TRPV4 is involved in OGD induced cell swelling, however, not necessarily through the activation of AQP4 channels. Several studies implied that TRPV4 collaborates with AQP4 channels in the process of RVD when non-physiological osmotic gradients are applied externally (Liedtke and Friedman, 2003; Ryskamp et al., 2014; Jo et al., 2015), but not in the case of electrical stimulation-induced cell swelling (Toft-Bertelsen et al., 2017). A similar difference between the TRPV4^–/–^ and AQP4^–/–^ animals was seen in the rodent stroke model, where the deletion of TRPV4 ameliorated peri-infarct depolarization (reduced influx of Ca^2+^ and lower extracellular accumulation of glutamate), however, the deletion of AQP4 did not have a similar effect (Rakers et al., 2017). It implies stronger involvement of neuronal rather than glial swelling. Furthermore, it was previously shown that TRPV4 activation following stroke increases NMDA receptor function, which may facilitate glutamate excitotoxicity (Li et al., 2013). Contradictory to the findings that detected slower changes of brain diffusivity in AQP4^–/–^ during acute ischemia *in vivo* (Yao et al., 2015; Hirt et al., 2017; Chmelova et al., 2019), we did not observe a similar influence of AQP4 deletion during OGD *in situ* (Chmelova et al., 2019). This could be associated with the previously seen protective effect of hypothermic (32–33°C) preconditioning that is present *in situ* (Jiang et al., 2018; Kreisman et al., 2020). Moreover, we previously demonstrated that severe pathological conditions are needed to reveal the role of AQP4 in cell swelling (Dmytrenko et al., 2013; Anderova et al., 2014). It is, therefore, possible that in the OGD model with a small oxygen content and removal of toxic metabolites by perfusion, the effect of AQP4 channel deletion may be beyond the discriminative ability of the RTI method.

TRPV4 deletion resulted in a partial protective effect during OGD manifested as a smaller decrease of α as compared to the Ctrl mice (Supplementary Table 2; Chmelova et al., 2019). In this study, OGD application in AQP4^–/–^/TRPV4^–/–^ mice did not result in any α decrease, which is in full agreement with the early phase of *in vivo* experiments, where the lesion area in the double knockouts is markedly reduced 1 day after pMCAO. The potentiating effect of the deletion of both TRPV4 and AQP4 channels may be explained by their mutual influence. It was shown that the protective effect of TRPV4^–/–^ deletion could be associated with the lower expression of AQP4 (Jo et al., 2015). Therefore, the complete deletion of both channels could have a more distinctive protective effect than the deletion of TRPV4 and merely a downregulation of the AQP4 expression.

The changes of the ECS diffusion parameters during OGD treatment in different strains correspond with the changes of [K^+^]_o_. While in the AQP4^–/–^ or TRPV4^–/–^ mice, the levels of [K^+^]_o_ did not differ from those in the Ctrl mice, the peak values during OGD in the AQP4^–/–^/TRPV4^–/–^ mice were significantly smaller. This finding suggests a role of neuronal activation due to TRPV4 deletion, as well as a contribution of impaired K^+^ channels functions due to AQP4 deletion. Shibasaki et al. (2007, 2015) showed that the influx of cations through TRPV4 in neurons may control neuronal excitability by regulating the resting membrane potential. The deletion of AQP4, which co-localizes with Kir4.1 channels, reduces the [K^+^]_o_ elevation in the AQP4^–/–^ mice as well as in α-syn^–/–^ mice (Amiry-Moghaddam et al., 2003b,Dmytrenko et al., 2013). In comparison with the Ctrl, we did not observe a significant difference in an increase of the [K^+^]_o_ during OGD between the AQP4^–/–^ or TRPV4^–/–^ and Ctrl mice. It is plausible that the concomitant deletion of both the AQP4 and TRPV4 channels was necessary to increase this difference to a significant level.

The increase of [K^+^]_o_ during OGD treatment in the control animals reached the maximum of about 8 mM, which is rather low compared to ischemia *in vivo* with a typical rise in [K^+^]_o_ exceeding 70 mM (Vorisek and Sykova, 1997; Chmelova et al., 2019). As aforementioned, the OGD model of ischemia in tissue slices cannot fully mimic real ischemic conditions *in vivo*, as it allows the removal of the toxic metabolites, and the perfusing solutions contain 5% of oxygen. Other studies have certainly also reported a much lower increase of [K^+^]_o_ during OGD than in ischemia *in vivo* (Rice and Nicholson, 1991; Perez-Pinzon et al., 1995). Moreover, the magnitude of [K^+^]_o_ increase considerably varied depending on the specific experimental conditions during OGD in these studies.

### The deletion of AQP4 or TRPV4 does not affect changes in the extracellular space diffusion parameters evoked by hypoosmotic stress or hyperkalemia

Even though fast osmotic changes in the tissue occur very rarely in reality, the model of hypoosmotic stress is useful in determining the alterations in cell volume regulation *in situ* (Toft-Bertelsen et al., 2018). Our previous studies in α-syn^–/–^ mice (Dmytrenko et al., 2013; Anderova et al., 2014) showed that a 30-min application of aCSF_H–50_ was not sufficient to differentiate between α-syn^–/–^ and the Ctrl mice, neither in α nor in astrocyte volume changes. On the other hand, aCSF_H–100_ resulted in a significantly smaller α decrease as well as a smaller astrocyte volume increase in the α-syn^–/–^ mice (Dmytrenko et al., 2013; Anderova et al., 2014). In contrast to our study, Thrane et al. (2011) observed a reduced swelling in the AQP4^–/–^ astrocytes when compared to the Ctrl ones, after exposure to only mild hypoosmotic stress but not after a more severe one. This discrepancy could be explained by the differences in glial homeostatic functions in different stages of maturity, as Thrane’s results are from 10- to 20-day old mouse pup slices. Since the AQP4 channels are highly permeable to water in the presence of high osmotic gradients (Ryskamp et al., 2014; Mola et al., 2016), the water permeability of astrocytes in AQP4 knockout (Solenov et al., 2004) or knock-down mice (Nicchia et al., 2003) is reduced by only about 50%. Presumably, other membrane proteins, such as glutamate transporters or other co-transporters (MacAulay et al., 2004; Kahle et al., 2015), participate in cell swelling and volume regulation.

In this study, we did not observe any difference in α after a 20-min application of aCSF_H–50_ or aCSF_H–100_ either in the AQP4^–/–^, TRPV4^–/–^, or AQP4^–/–^/TRPV4^–/–^ mice, compared to the Ctrl. Of note, the differences in α that we described previously between the α-syn^–/–^ mice and the Ctrl, during the application of aCSF_H–100_, were not distinguishable before the 20th minute of application either. In contrast, the volume of single astrocytes differed as early as the 10th minute of exposure to hypoosmotic stress (Anderova et al., 2014). We can assume that the volume regulation in astrocytes precedes the ECS volume changes, and that longer hypoosmotic stimulation might be required to detect the more pronounced effect of AQP4 deficiency. Moreover, since the lower temperature than the physiological one led to an increase in surface localization of AQP4 in human astrocytes (Salman et al., 2017), the different temperature conditions used in the current (close to physiological temperature) and previous (room temperature) studies could also explain the discrepancy in the results. In several studies of cell cultures lacking TRPV4 or AQP4, the results are inconsistent (Benfenati et al., 2011; Jo et al., 2015; Pivonkova et al., 2018). We did not observe RVD in any experimental group in our study. However, several studies indicated that RVD, as described in cell cultures, was mostly absent *in situ* (Pivonkova et al., 2018). The slower swelling kinetics also seen in the AQP4^–/–^ and TRPV4^–/–^ were observed within the 1-min range (Jo et al., 2015). We are not able to detect changes in α in such short intervals with the RTI method.

Due to the co-localization of AQP4 with Kir4.1, functional coupling between these proteins has been proposed (Nagelhus et al., 1999; Amiry-Moghaddam et al., 2003a). Previous research on α-syn^–/–^ mice revealed reduced astrocyte swelling induced by the application of a 50 mM K^+^ solution, but not by the application of milder 10 mM K^+^ solution (Anderova et al., 2014), suggesting the important role of AQP4 in K^+^ homeostasis under pathological conditions. The reciprocal regulation of the gene expression of Trpv4, Aqp4 and Kir4.1 has also been described. It has further been shown in the mouse retina that the deficiency of both AQP4 and TRPV4, leads to the reduced expression of Kir4.1 (Jo et al., 2015). Their findings are in agreement with lower [K^+^]_o_ in this study. Interestingly, we report no difference in α changes after a 20-min application of 50 mM K^+^ solution, either in the AQP4^–/–^, TRPV4^–/–^,or AQP4^–/–^/TRPV4^–/–^ mice as compared with the Ctrl. This may be explained by the increased expression of astrocytic gap junctions that occurs due to the lack of AQP4 (Katoozi et al., 2017), leading to more efficient K^+^ buffering. The protective effect of the interplay between AQP4 and Kir 4.1 was also shown by Posati et al. (2016), while other studies denied functional coupling of AQP4 and Kir 4.1 channels (Zhang and Verkman, 2008).

## Conclusion and hypotheses

**FIGURE 5:**
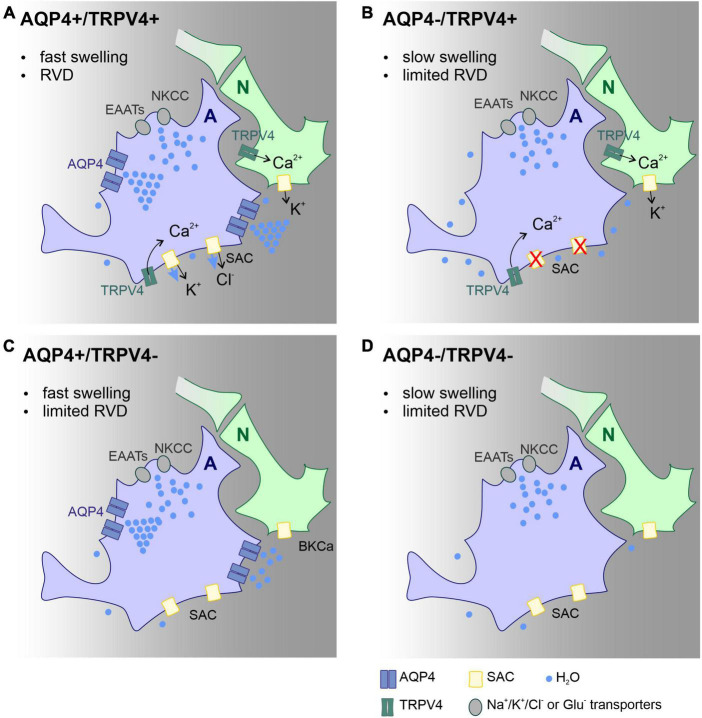
Scheme of the main mechanisms involved in astrocyte volume changes and volume regulation in the acute post-ischemic phase, effect of AQP4 and TRPV4 channel deletion. Proposed mechanisms explaining the changes in the lesion size observed in the Ctrl **(A)**, AQP4^–/–^ **(B)**, TRPV4^–/–^ **(C)**, and AQP4^–/–^/TRPV4^–/–^ **(D)** mice. We propose that the absence of AQP4 in single **(B)** or double knock-outs **(D)** leads to slowed swelling that is insufficient to trigger regulatory volume decrease (RVD). Such a decrease in RVD presumably occurs due to low activity of TRPV4 channels, which is unable to activate SACs in panel **(B)**, or lack of TRPV4 channels in panel **(D)**. Water entry into the cell occurs *via* ion/glutamate transporters, while its efflux occurs besides ion channels by simple diffusion. Deletion of TRPV4 results in the absence of RVD activated by cell membrane stretch. Both slower swelling and limited RVD in double knock-outs, lead to less edema in the acute post-ischemic phase compared to control. Panels **(B,D)** both display limited swelling as well as RVD however in AQP4^–/–^ **(B)** functional TRPV4 channels in neurons prolong neuronal activity with further neurotoxic impact on nervous tissue. A, astrocyte; N, neuron; Ctrl, control; AQP4^–/–^, AQP4-deficient mice; TRPV4^–/–^, TRPV4-deficient mice; AQP4^–/–^/TRPV4^–/–^, AQP4- and TRPV4-deficient mice; RVD, regulatory volume decrease; NKCC, Na^+^/K^+/^Cl^–^ co-transporter; SAC, stretch activated ion channel; EAAT, excitatory amino acid transporter; AQP4, aquaporin-4 channel; TRPV4, Transient receptor potential cation channel subfamily V member 4.

Our findings add to a growing body of literature on the functional involvement of the AQP4/TRPV4 complex in pathological cell swelling. This is the first study that describes the impact of AQP4/TRPV4 deletion on the size of the ischemic lesion (MRI) and ECS diffusion parameters in experimental models of ischemia, OGD, hyperkalemia, and hypotonic stress in AQP4^–/–^/TRPV4^–/–^ mice. The results obtained in this study indicate that the interplay between AQP4 and TRPV4 channels may play a crucial role in the size of early edema development following ischemia. Protective effect of simultaneous deletion of AQP4 and TRPV4 in development of acute ischemia (OGD model) and in early phase following ischemia *in vivo* is highly probably a complex mechanism involving functional alteration of several cell types, including astrocytes, neurons, endothelial cells and microglia. Presumably, the most important mechanism in the acute post-ischemic phase is alteration of astrocytic swelling as suggested by Mola et al. (2016). According to their model, speed of water influx in the absence of AQP4 is decreased and results in slow cell swelling that is incapable to trigger RVD, a process leading to cell volume restoration. Thus, smaller lesion observed in double knock-out animals in the acute phase after MCAO dwells primarily from slower swelling of astrocytes. In addition, this protective effect may be further potentiated by a lower neuronal excitability and lower extracellular potassium concentration in the absence of TRPV4. On the contrary, inefficient RVD and involvement of microglia activation may play a role in the worsened outcome in late (chronic) post-ischemic phase, as both AQP4 and TRPV4 are involved in regulation of microglial activity and post-ischemic inflammation. For details see schematic representation of this hypothesis (Figure 5).

Moreover, it is highly probable that besides AQP4, the TRPV4 channel might collaborate with other effector proteins. This research has given rise to many questions in need of further investigation to reveal new targets for potential therapeutic interventions.

## Data availability statement

The raw data supporting the conclusions of this article will be made available by the authors, without undue reservation.

## Ethics statement

All procedures involving the use of laboratory animals were performed in accordance with the Council Directive 2010/63EU of the European Parliament and the Council of 22 September 2010, on the protection of animals used for scientific purposes and animal care guidelines approved by the Institute of Experimental Medicine, Academy of Sciences of the Czechia (Animal Care Committee on 7 April 2011; approval number 49/2019).

## Author contributions

PS and ZH analyzed and interpreted the data, wrote the manuscript, and prepared the figures. PS, ZH, and MC cross-bred the experimental mice. PS and MC performed the real-time iontophoretic method. DK and SC performed the pMCAO surgery. ES and DJ performed the MRI and processed and analyzed the data. IV performed DW-MRI. ZH and VM performed the IHC. JT, LV, and MA revised and edited the manuscript. LV and MA conceived and supervised the study. All authors contributed to the article and approved the submitted version.

## Footnotes

1
http://www.cdb.riken.jp/arg/mutant%20mice%20list.html

2
https://www.wpiinc.com/ie190-potassium-ion-exchanger
