# Supplementary figures and images for: The absence of AQP4/TRPV4 complex substantially reduces acute cytotoxic edema following ischemic injury
Source: Front Cell Neurosci. 2022 Dec 8;16:1054919. doi: 10.3389/fncel.2022.1054919 (PMC9773096; doi:10.3389/fncel.2022.1054919)

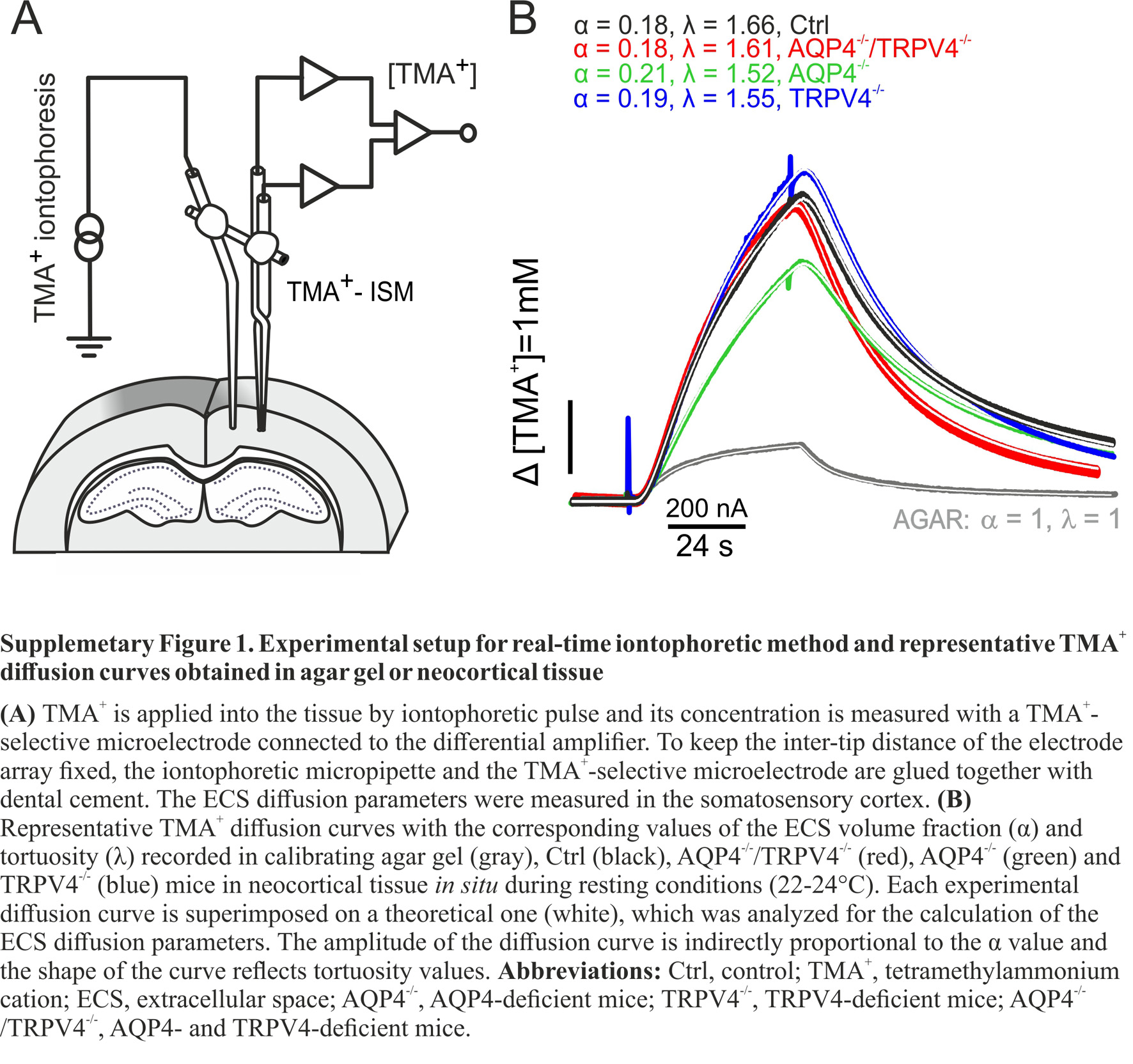

Supplement: Supplementary file 1 [file Image_1.jpg]
