# Supplementary material for: The absence of AQP4/TRPV4 complex substantially reduces acute cytotoxic edema following ischemic injury
Source: Front Cell Neurosci. 2022 Dec 8;16:1054919. doi: 10.3389/fncel.2022.1054919 (PMC9773096; doi:10.3389/fncel.2022.1054919)
Supplement: Supplementary file 2 [file Table_1.DOCX]

**Supplemetary Table 1:** Absolute values of the extracellular volume fraction (α), tortuosity (λ), and non-specific uptake (kʹ) in the somatosensory cortex of AQP4^‑/-^/TRPV4^-/-^, AQP4^-/-^ and TRPV4^-/-^ mice and of their age-matched Ctrl during 20^th^ min of hypoosmotic stress (aCSF_H‑50_, aCSF_H‑100_), OGD and hyperkalemia (aCSF_K+_). Data are expressed as mean ± SEM, N represents the number of animals in the group, n represents the number of individual applications.

|  | **Control** | | **AQP4^-/-^/TRPV4^-/-^** | | **TRPV4^-/-^** | | | | **AQP4^-/-^** | | |
| --- | --- | --- | --- | --- | --- | --- | --- | --- | --- | --- | --- |
|  | ***aCSF*** | **aCSF_H-50_** | ***aCSF*** | **aCSF_H-50_** | ***aCSF*** | **aCSF_H-50_** | | | ***aCSF*** | | **aCSF_H-50_** |
| α | 0.201 ± 0.004 | 0.140 ± 0.008 | 0.190 ± 0.006 | 0.149 ± 0.01 | 0.195 ± 0.002 | 0.143 ± 0.006 | | 0.218 ± 0.002 | | | 0.161 ± 0.008 |
| λ | 1.654 ± 0.022 | 1.650 ± 0.038 | 1.610 ± 0.019 | 1.722 ± 0.036 | 1.572 ± 0.020 | 1.645 ± 0.026 | | 1.609 ± 0.014 | | | 1.681 ± 0.029 |
| kʹ  [10^-3^s^-1^] | 14.9 ± 2.4 | 11.8 ± 2.5 | 17.5 ± 3.9 | 21.7 ± 2.6 | 0.13 ± 1.84 | 16.8 ± 2.5 | | 0.13 ± 2.02 | | 12.9 ± 2.3 | |
| N/n | 6/10 | | 8/9 | | 6/14 | | 6/11 | | | | |

|  | **Control** | | **AQP4^-/-^/TRPV4^-/-^** | | **TRPV4^-/-^** | | | | **AQP4^-/-^** | | |
| --- | --- | --- | --- | --- | --- | --- | --- | --- | --- | --- | --- |
|  | ***aCSF*** | **aCSF_H-100_** | ***aCSF*** | **aCSF_H-100_** | ***aCSF*** | **aCSF_H-100_** | | | ***aCSF*** | | **aCSF_H-100_** |
| α | 0.187 ± 0.004 | 0.103 ± 0.007 | 0.202 ± 0.006 | 0.101 ± 0.009 | 0.196 ± 0.004 | 0.113 ± 0.010 | | 0.214 ± 0.008 | | | 0.108 ± 0.009 |
| λ | 1.601 ± 0.017 | 1.636 ± 0.024 | 1.619 ± 0.016 | 1.706 ± 0.035 | 1.595 ± 0.018 | 1.682 ± 0.020 | | 1.635 ± 0.007 | | | 1.705 ± 0.030 |
| kʹ  [10^-3^s^-1^] | 19.1 ± 6.7 | 15.3 ± 6.5 | 14.1 ± 2.4 | 12.6 ± 2.6 | 10.6 ± 2.4 | 10.7 ± 2.3 | | 8.86± 1.72 | | 8.3 ± 1.2 | |
| N/n | 8/9 | | 10/12 | | 7/9 | | 10/12 | | | | |

|  | **Control** | | **AQP4^-/-^/TRPV4^-/-^** | | **TRPV4^-/-^** | | | | **AQP4^-/-^** | | |
| --- | --- | --- | --- | --- | --- | --- | --- | --- | --- | --- | --- |
|  | ***aCSF*** | ***OGD*** | ***aCSF*** | ***OGD*** | ***aCSF*** | ***OGD*** | | | ***aCSF*** | | ***OGD*** |
| α | 0.197 ± 0.005 | 0.107 ± 0.004 | 0.197 ± 0.005 | 0.207 ± 0.009 | 0.196 ± 0.005 | 0.141 ± 0.009 | | 0.200 ± 0.007 | | | 0.109 ± 0.008 |
| λ | 1.619 ± 0.02 | 1.651 ± 0.034 | 1.601 ± 0.025 | 1.646 ± 0.047 | 1.631 ± 0.02 | 1.647 ± 0.039 | | 1.610 ± 0.018 | | | 1.621 ± 0.034 |
| kʹ  [10^-3^s^-1^] | 12.7 ± 2.6 | 7.94 ± 2.4 | 15.3 ± 4.1 | 8.63 ± 2.5 | 11.8 ± 2.2 | 15.0 ± 5.6 | | 8.32 ±1.8 | | 5.1 ± 0.9 | |
| N/n | 11/14 | | 8/8 | | 7/9 | | 10/12 | | | | |

|  | **Control** | | **AQP4^-/-^/TRPV4^-/-^** | | **TRPV4^-/-^** | | | | **AQP4^-/-^** | | |
| --- | --- | --- | --- | --- | --- | --- | --- | --- | --- | --- | --- |
|  | ***aCSF*** | **aCSF_K+_** | ***aCSF*** | **aCSF_K+_** | ***aCSF*** | **aCSF_K+_** | | | ***aCSF*** | | **aCSF_K+_** |
| α | 0.192 ± 0.006 | 0.123 ± 0.006 | 0.210 ± 0.005 | 0.118 ± 0.007 | 0.190 ± 0.004 | 0.129 ± 0.008 | | 0.214 ± 0.005 | | | 0.144 ± 0.008 |
| λ | 1.583 ± 0.013 | 1.710 ± 0.044 | 1.584 ± 0.023 | 1.704 ± 0.048 | 1.654 ± 0.025 | 1.770 ± 0.041 | | 1.618 ± 0.023 | | | 1.731 ± 0.036 |
| kʹ  [10^-3^s^-1^] | 15.4 ± 2.2 | 13.3 ± 4.2 | 12.0 ± 3.0 | 9.66 ± 3.1 | 12.2 ± 2.5 | 9.10 ± 1.7 | | 12.2 ± 1.4 | | 11.2 ± 2.0 | |
| N/n | 9/13 | | 9/10 | | 7/11 | | 7/11 | | | | |
